# Supplementary material for: Auditory Processing and Speech Sound Disorders: Behavioral and Electrophysiological Findings
Source: Audiol Res. 2025 Sep 19;15(5):119. doi: 10.3390/audiolres15050119 (PMC12452735; doi:10.3390/audiolres15050119)

Comparative Neurophysiological Measures between TD and SSD Groups

Results

The results of the neurophysiological measures (ABR, MLR, LLR; latencies) are presented below.

Table S1. ABR Latencies (TD vs SSD)

GROUP	ABR_RIGHT_IPSILAT_V_MS	ABR_RIGHT_IPSILAT_I_V_MS	ABR_RIGHT_CONTROLAT_V_MS	ABR_RIGHT_CONTROLAT_I_V_MS	ABR_LEFT_IPSILAT_V_MS	ABR_LEFT_IPSILAT_I_V_MS	ABR_LEFT_CONTROLAT_V_MS	ABR_LEFT_CONTROLAT_I_V_MS
SSD	6.03 ± 0.25	0.36 ± 0.1	6.16 ± 0.21	0.27 ± 0.08	5.99 ± 0.24	0.35 ± 0.12	6.08 ± 0.32	0.25 ± 0.09
TD	6.09 ± 0.23	0.43 ± 0.13	6.18 ± 0.21	0.3 ± 0.12	6.03 ± 0.23	0.42 ± 0.09	6.08 ± 0.19	0.3 ± 0.16

Figure S1. ABR Wave V Latencies (TD vs SSD)

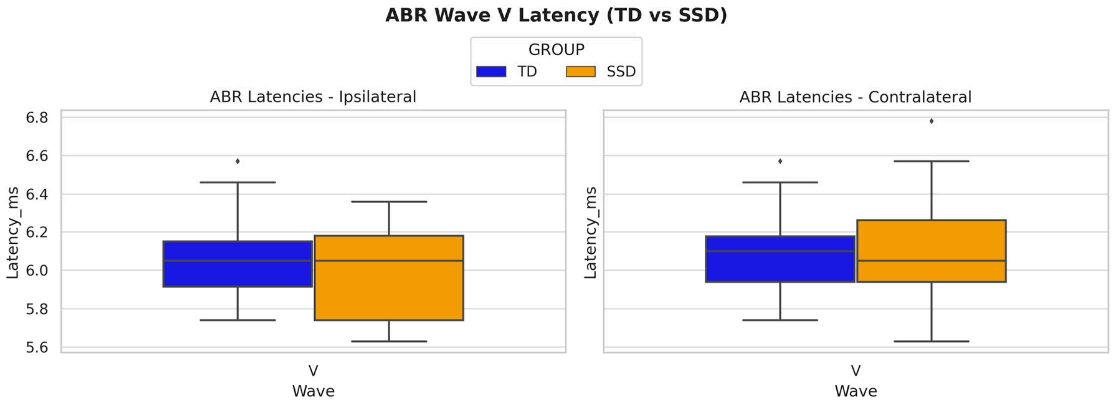

Figure S2. ABR I-V wave Latencies (TD vs SSD)

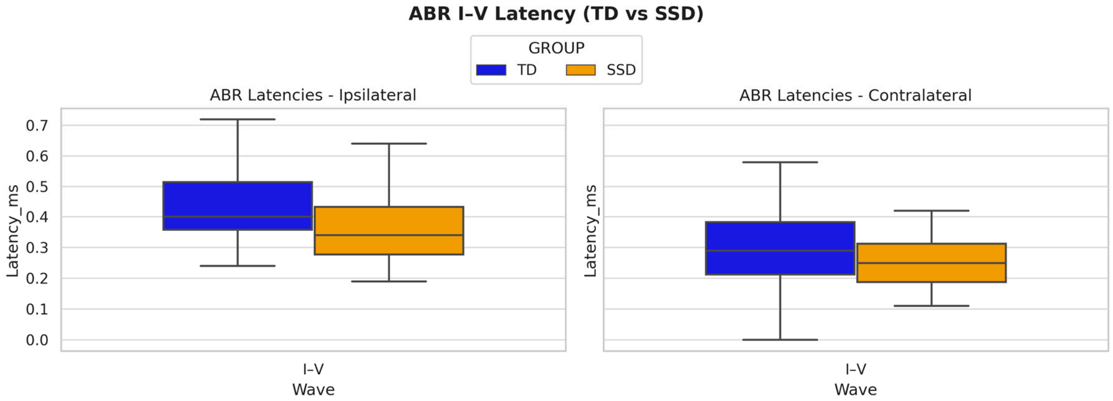

Figure S3. SSD ABR Waveforms

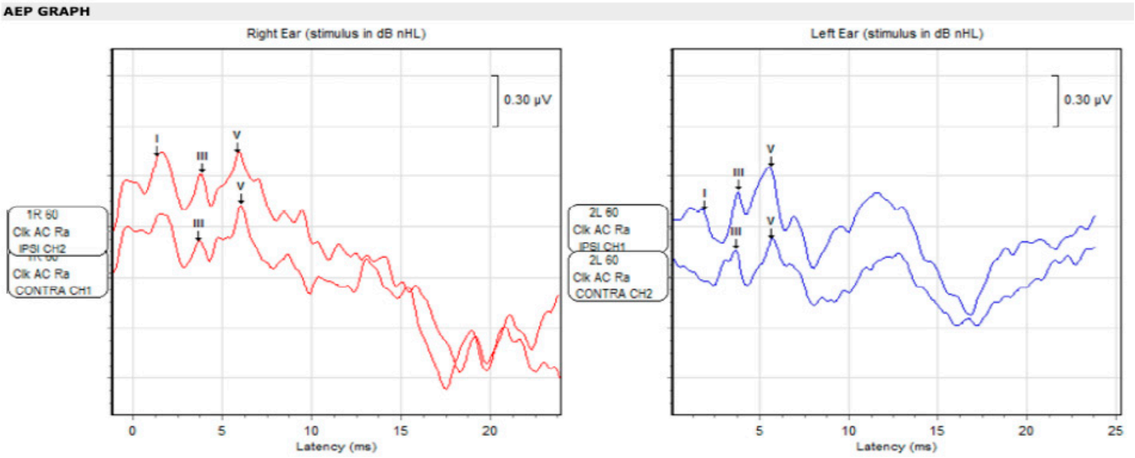

Figure S4. TD ABR Waveform

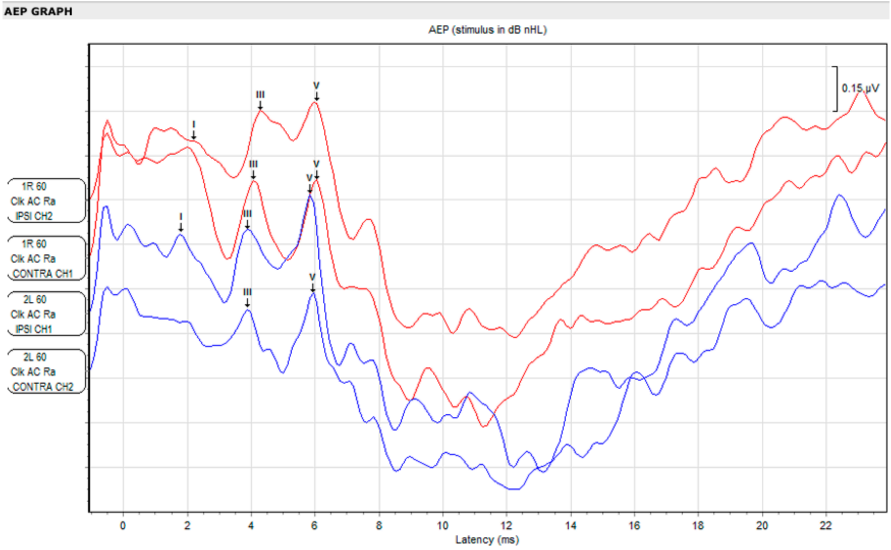

Table S2. MLR Latencies (TD vs SSD)

GROUP	MLR_RIGHT_IPSILAT_TIME_Na	MLR_RIGHT_IPSILAT_TIME_Pa	MLR_RIGHT_CONTROLAT_TIME_Na	MLR_RIGHT_CONTROLAT_TIME_Pa	MLR_LEFT_IPSILAT_TIME_Na	MLR_LEFT_IPSILAT_TIME_Pa	MLR_LEFT_CONTROLAT_TIME_Na	MLR_LEFT_CONTROLAT_TIME_Pa
SSD	30.01 ± 3.2	37.15 ± 4.38	29.82 ± 3.04	36.94 ± 4.75	27.11 ± 4.3	36.89 ± 5.28	28.47 ± 6.83	36.82 ± 7.07
TD	30.67 ± 4.73	39.28 ± 5.88	29.6 ± 4.94	37.52 ± 5.76	29.72 ± 4.34	37.54 ± 4.12	29.47 ± 5.51	38.3 ± 5.22

MLR Measures

Figure S5. MLR Na Latencies (TD vs SSD)

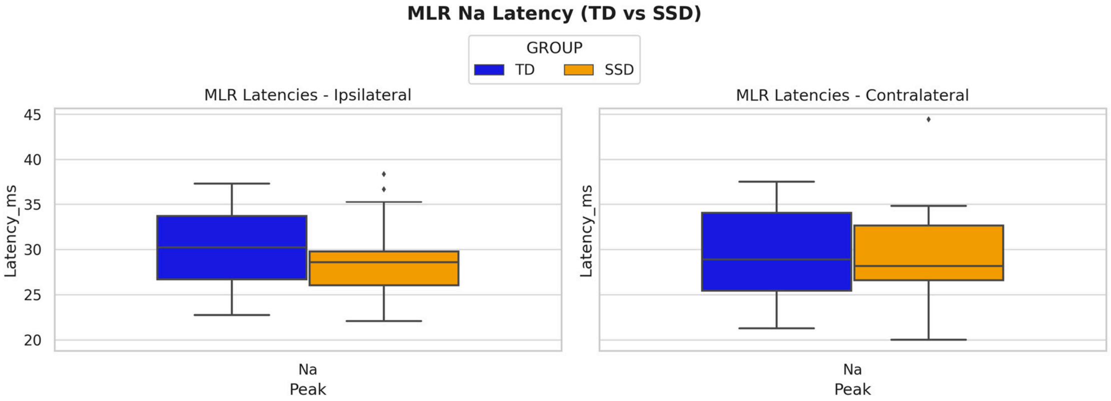

Figure S6. MLR Pa Latencies (TD vs SSD)

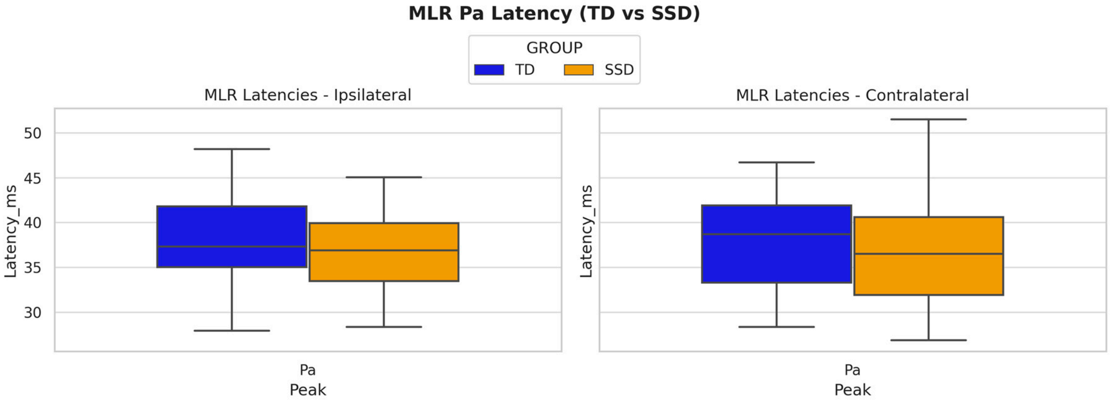

Figure S7. SSD MLR Waveform

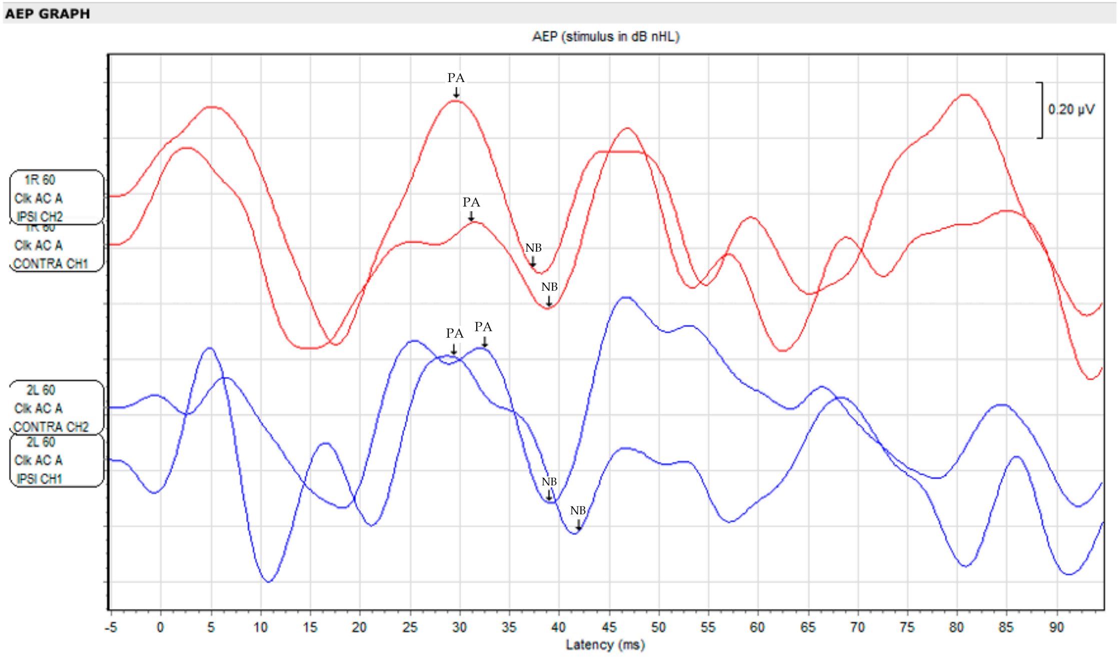

Figure S8. TD MLR Waveform

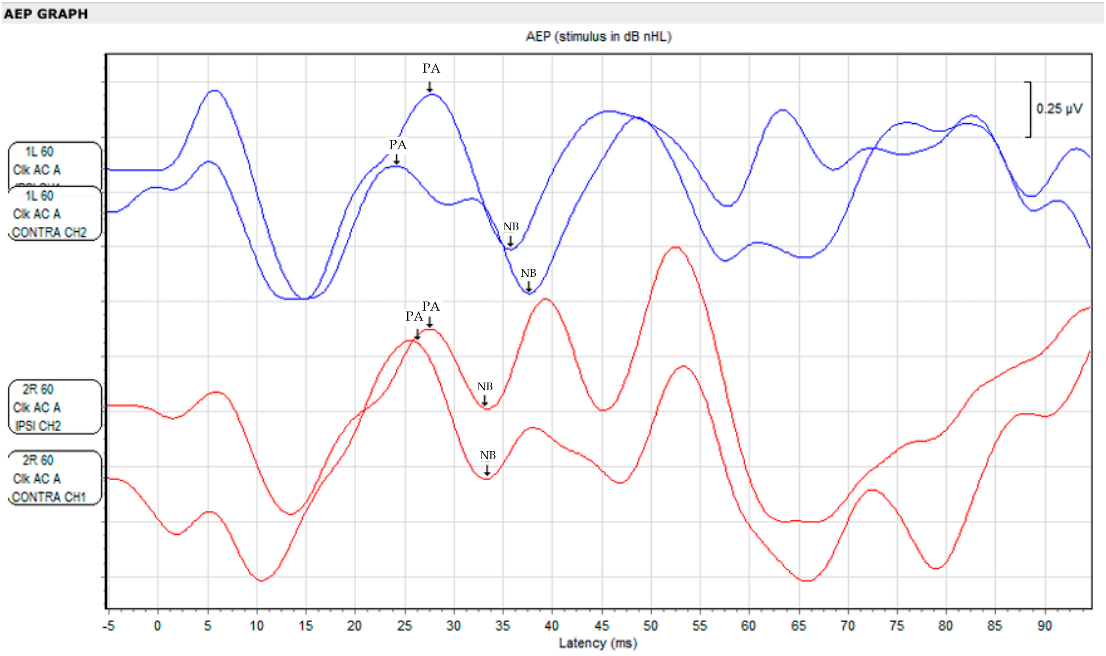

Table S3. LLR Latency (TD vs SSD)

GROUP	LLR_RIGHT_IPSILAT_TIME_P1	LLR_RIGHT_IPSILAT_TIME_P2	LLR_RIGHT_CONTROLAT_TIME_P1	LLR_RIGHT_CONTROLAT_TIME_P2	LLR_LEFT_IPSILAT_TIME_P1	LLR_LEFT_IPSILAT_TIME_P2	LLR_LEFT_CONTROLAT_TIME_P1	LLR_LEFT_CONTROLAT_TIME_P2
SSD	91.07 ± 39.84	197.73 ± 42.97	89.4 ± 32.83	193.01 ± 45.0	81.21 ± 33.45	177.04 ± 48.69	78.57 ± 34.83	181.34 ± 46.09
TD	71.41 ± 15.36	171.41 ± 34.87	72.84 ± 14.39	171.68 ± 39.5	72.72 ± 13.44	166.88 ± 48.11	73.67 ± 11.01	173.16 ± 45.79

LLR Measures

Figure S9. LLR P1 Peak Latencies (TD vs SSD)

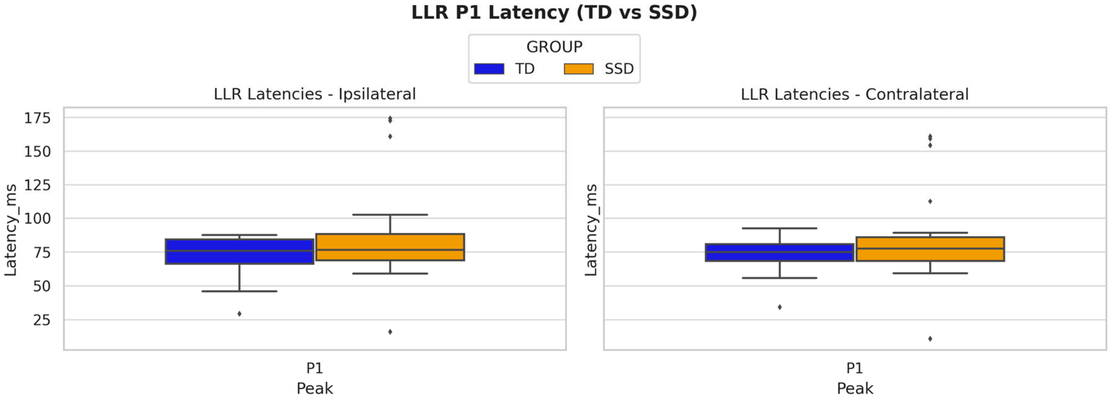

Figure S10. LLR P2 Pick Latencies (TD vs SSD)

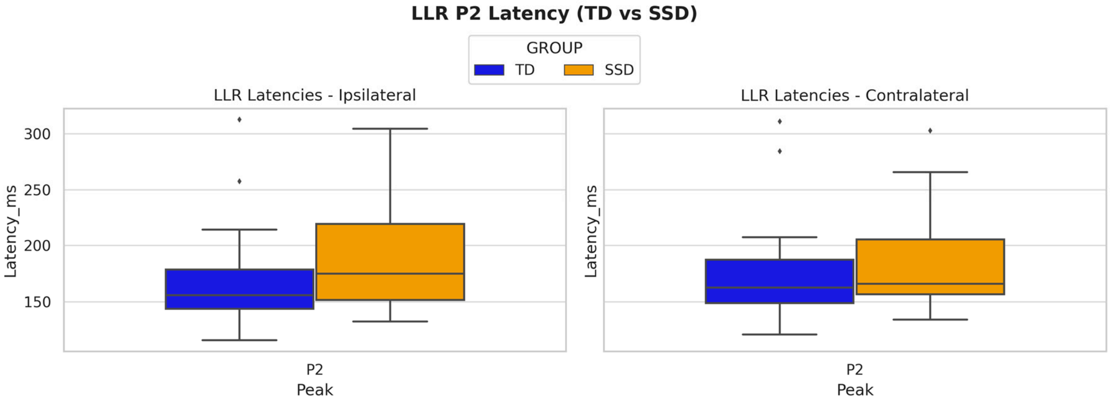

Figure S11. SSD LLR Waveform

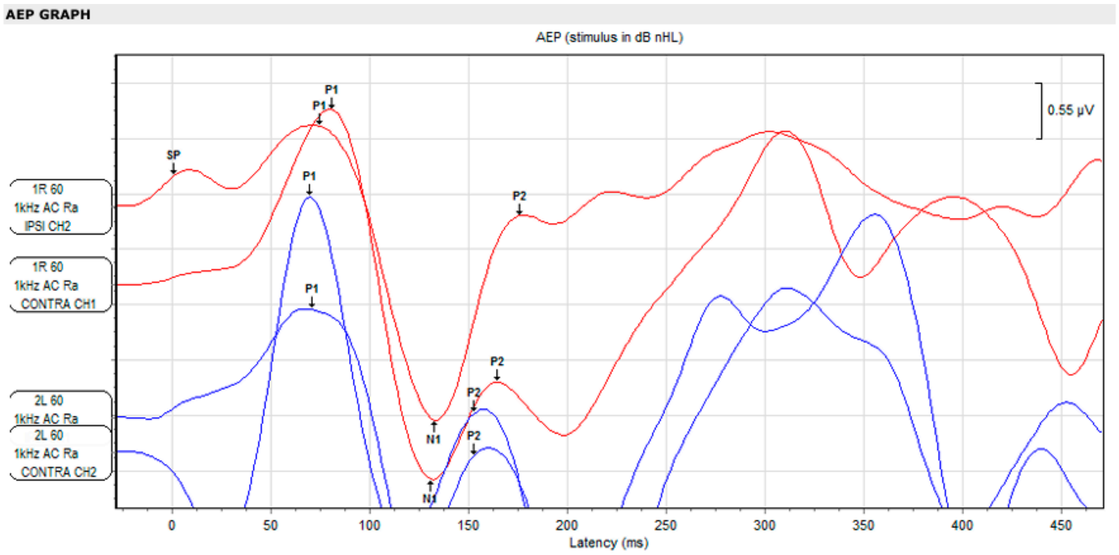

Figure S12. TD LLR Waveform

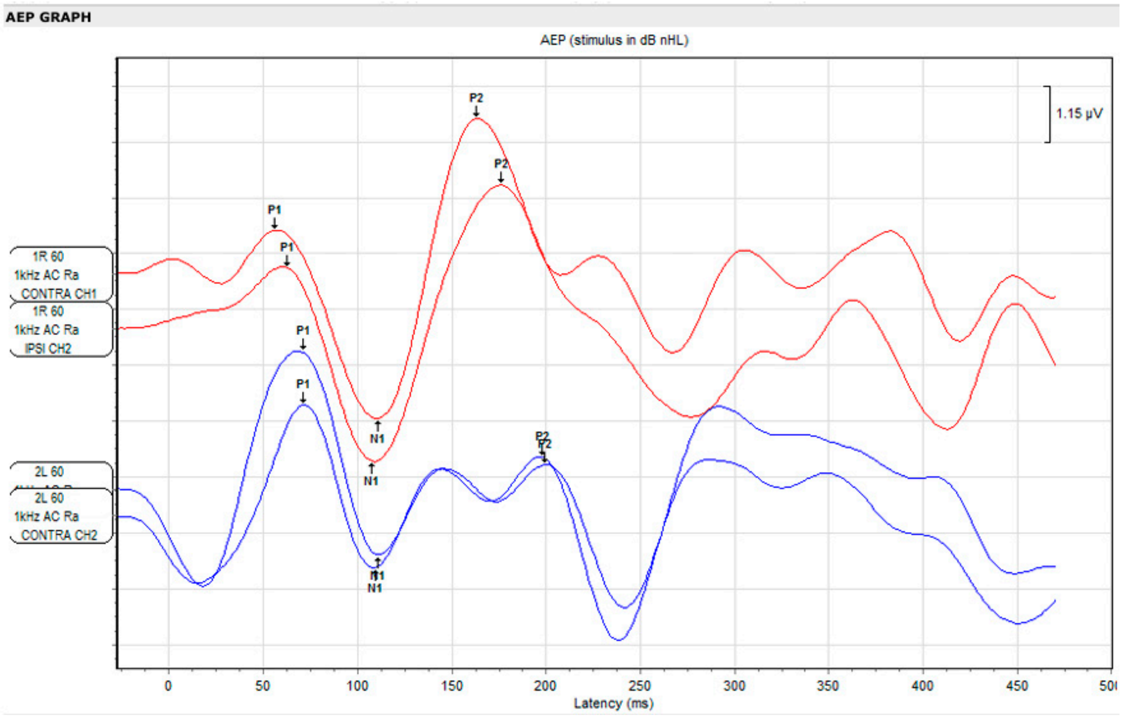

Supplement: Supplementary file 1 [file audiolres-15-00119-s001.zip › audiolres-3259258-supplementary.pdf]
